# Mystery of Hepatitis E Virus: Recent Advances in Its Diagnosis and Management

**DOI:** 10.1155/2015/872431

**Published:** 2015-01-19

**Authors:** Aftab Ahmed, Ijlal Akbar Ali, Hira Ghazal, Javid Fazili, Salman Nusrat

**Affiliations:** ^1^Department of Internal Medicine, Oklahoma University Health Sciences Center, Williams Pavilion 1130, P.O. Box 26901, Oklahoma City, OK 73104, USA; ^2^Dow Medical College, Mission Road, Karachi 74200, Pakistan; ^3^Section of Digestive Diseases and Nutrition, Oklahoma University Health Sciences Center, Williams Pavilion 1345, 920 SL Young Boulevard, Oklahoma City, OK 73104, USA

## Abstract

Mysterious aspects of the long presumed to be well-known hepatitis E virus (HEV) have recently surfaced that distinguish it from other hepatotropic viruses. It is a cause of chronic hepatitis in immunosuppressed patients. It has human to human transmission through blood and mantains high seroprevalence in blood donors. HEV has also been found to occur more frequently in the West in those without a history of travel to endemic countries. It has varied extrahepatic manifestations and has multiple non-human reservoirs including pigs and rats. Considering these recent discoveries, it appears odd that HEV is not sought more frequently when working up acute and chronic hepatitis patients. The disease is particularly severe among pregnant women and has a high attack rate in young adults. What adds to its ambiguity is the absence of a well-established diagnostic criteria for its detection and that there is no specific antiviral drug for hepatitis E, except for isolated cases where ribavirin or pegylated interferon alpha has been used with occasional success. This review paper discusses the recent advances in the knowledge of the virus itself, its epidemiology, diagnostic approach and prevention, and the treatment options available.

## 1. Introduction

In 1978 the existence of a non-A, non-B hepatitis virus, likely hepatitis E virus (HEV) was suggested during an outbreak of acute hepatitis in Kashmir [1–3]. However, it was not truly identified until 1983 when a researcher investigating an outbreak of unexplained hepatitis in Soviet soldiers in Afghanistan ingested fecal extract from affected military personnel and developed acute hepatitis and small viral particles were identified in his stool [3]. Recently, mysterious aspects of this once presumed to be well known virus have surfaced. Over the last few years our practices and understanding pertaining to its prevalence, mode of infection, clinical manifestations, diagnostic tests, treatment options, and role of vaccination have evolved tremendously. There is now good evidence that HEV infection is neither rare nor limited to developing countries and no clinical presentation is limited to acute infection (Table 2). Lack of knowledge among physicians and absence of standardized tests result in failure to diagnose and therefore can lead to an increase in morbidity and mortality. All this underscores the importance of HEV as a virus that might have taken us off guard.

## 2. Virology and Taxonomic Status

**Figure 1:**
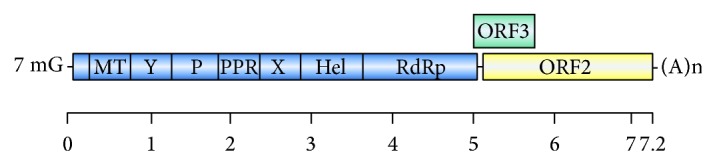
The structure of the hepatitis E virus genome. RNA length: 7.2 kb. It has short 5 and 3 noncoding regions and three overlapping open reading frames (ORFs). ORF1 encodes the nonstructural proteins, including a methyl transferase (MT), cysteine protease (P), helicase (Hel), and RNA polymerase (RdRp), as well as three regions of unknown function (Y, PPR, and X). The 5 end of the RNA genome is capped with 7-methylguanosine (7 mG), and the 3 end is polyadenylated (poly A).

HEV is a small (27–34 nm) nonenveloped virus. The viral genome consists of a single-stranded, positive-sense RNA molecule organized into three open reading frames (ORF1, ORF2, and ORF3) (Figure 1). ORF1 is involved in viral replication and protein processing through RNA-dependent RNA polymerase. ORF2 encodes the viral capsid protein, which is involved in attachment to host cells and induction of neutralizing antibodies. Finally, ORF3 encodes for a small immunogenic phosphorylated protein (pORF3) involved in virion morphogenesis and release [4].

It belongs to the Hepeviridae family and is the sole member of genus* Hepevirus* [5]. HEV genotypes are further classified into subtypes: genotype 1, five (1a–1e); genotype 2, two (2a and 2b); genotype 3, ten (3a–3j); and genotype 4, seven (4a–4g) [4]. Genotype 2 appears to be exclusively anthroponotic, while genotype 1 infects mainly humans but has also been detected in pigs [4, 6]. Genotypes 3 and 4 also infect other animals, particularly pigs (Table 1).

## 3. Epidemiology

HEV is one of the leading causes of hepatitis worldwide. Human infection with HEV is not only prevalent in developing countries but is also common in western nations and based on geographical distribution there are two distinct epidemiological patterns [3, 7].

### 3.1. Infection in Developing Countries

Hepatitis E has long been considered a disease of developing countries. Although lack of routinely available diagnostics tests and dual infection with HAV makes it difficult to estimate its true prevalence, some have reported a prevalence as high as 50% [8]. Outbreak is more common during summer. For unclear reasons it predominantly affects males between the ages of 15 and 30 years and is uncommon in children younger than 10 years [3]. Genotype 1 tends to be more common in Southern and Central Asia; similarly others are more prevalent in other geographical locations (Table 1). HEV1 is prevalent in Southern and Central Asia, the Far East, North Africa, and the Caribbean. HEV2 is predominant in Mexico (probably subtype 2a) and West Africa (subtype 2b). HEV3 infections are found to occur worldwide, including America, Europe, China, and Japan and HEV4 predominates in China whilst also being detected in swine livestock from India and Indonesia and recently in Central Europe [1, 3, 6, 7, 9, 10]. Increasing the complexity of the HEV epidemiology, HEV is occasionally associated with hepatitis A virus outbreaks in developing regions in the form of dual infection [6]. These waterborne outbreaks have been exclusively associated with genotypes 1 and 2 strains [6]. Due to the lesser stability of its particle, HEV is less prevalent in the population than HAV [9].

### 3.2. Infection in Developed Countries

In developed countries, HEV infection has traditionally been recognized among travelers returning from endemic countries. However, during the past decade, sporadic autochthonous (locally acquired) cases of HEV genotypes 3 and 4 infection have also been reported without recent travel history to endemic countries [3, 11], with a predilection for middle-aged elderly males aged over 40 (mean age 60 years; male/female ratio = 3 : 1) [3]. In contrast to prevalence in developing parts of the world, in non-endemic countries hepatitis E represents about 1% of the acute viral hepatitis [7, 12]. As exposure appears to be unrelated to age or sex, it seems unconventional [13] and puzzling that older men are more often exposed to HEV and implies that host factors could be important [3]. HEV strains responsible for autochthonous infections have been isolated from various animal species including wild and domestic swine, deer, chicken, rats, rabbit [6], and camels [14], slurry lagoons, rivers, the sea, shellfish, and soft fruits [13]. Phylogenetic studies have proven the relationship between HEV strains circulating in pigs and human beings, thus promoting the notion of autochthonous zoonotic transmission [3].

## 4. Mode of Transmission

The fecooral route is the primary and most well documented mode of transmission. It is more prevalent with HEV-1 and -2 and explains the endemicity and frequent outbreaks of HEV-1 and -2 in developing countries [7, 15, 16]. Genotype 3 has been detected in pig liver in grocery stores and thus may partially account for HEV exposure in the United States [17].

In developed countries, some cases of vertical transmissions of HEV have been reported [4]. However, transmission through breast milk has not been described [6].

Recently transmission via blood transfusions has become a concern. Seroprevalence rates as high as 50% have been reported among asymptomatic individuals in nonendemic countries. Although not representative of active infection or carrier state, such high seropositive states in asymptomatic individuals raise concerns about the spread of infection during periods of infectivity particularly in the absence of standardized screening tests [4, 6].

HEV has recently been reported in homosexual men, which supports its sexual transmission [18, 19]. Direct animal transmission is also a possibility as studies have shown that veterinarians and swine handlers have more chances to be anti-HEV IgG positive than the general population [3].

## 5. Clinical Presentation

Hepatitis E has variable clinical presentations and ranges from asymptomatic carriers to fulminant hepatitis. As one would expect clinical manifestations to some extent depend on the predominant genotype. In endemic areas where genotypes 1 and 2 are most prevalent it primarily manifests as acute hepatitis. On the other hand in developed countries genotypes 3 and 4 are more prevalent and patients are mostly asymptomatic [25]. This notion was supported by Kumar et al. who identified that all the acute cases resulted from subtype Ia and that the fulminant cases were secondary to subtype Ic [16]. However, Smith and Simmonds [26] in their study suggested that it is the host-specific factors rather than virus genotype that dictate the risk of fulminant hepatitis.

In acute HEV, the incubation period is 3–8 weeks followed by a short prodromal phase. The symptomatic phase can last anywhere from days to several weeks (mean 4–6 weeks) [27]. As with acute hepatitis from other etiologies patients present with jaundice, right upper quadrant pain, and nondescript symptoms such as fever, asthenia, nausea, vomiting, and joint pains [1, 28, 29]. Recent data has shown that it is not uncommon that patients with acute hepatitis from HEV are misdiagnosed as having drug-induced liver injury [22]. HEV at times can infect patient with chronic liver disease; however a similar clinical picture makes diagnosis difficult and challenging [7, 13, 23, 30]. This is particularly important as prognosis is grim in patients with preexisting chronic liver disease and some studies report a mortality rate as high as 70% [3]. Other risk factors for acute liver failure are noted in Table 1.

A wide range of extrahepatic manifestations have been attributed to HEV. Those associated with acute illness include rash and arthralgia [24], Guillain-Barre syndrome [31, 32], myasthenia gravis [33], bilateral brachial neuritis, peripheral neuralgia with meningitis, seizures, nerve palsies, and pseudotumor cerebri.

### 5.1. Chronic Hepatitis

Chronic HEV infection by definition requires elevated aminotransferase levels, positive serum HEV RNA, and suggestive histologic findings for at least 3 months [3]. It is usually caused by genotype 3 and chronic infection secondary to genotypes 1 and 2 has not been documented [4]. One case of chronic HEV infection by genotype 4 has been reported in the literature [20]. Risk factors include immunosuppression, solid organ transplantation, HIV infection, hemodialysis, and hematological malignancies [1, 3, 4, 17, 34–40].

The route of transmission is not different from that seen in acute infection [3]. Most patients are either asymptomatic or present with vague symptoms. Extraintestinal manifestations neuralgic amyotrophy [41], peripheral neuropathies, encephalitis [32, 42], encephalopathy [32, 43], Parsonage-Turner syndrome [42], paroxysmal myopathy, and bilateral pyramidal syndromes are known to occur [1].

Presence of chronic infection in immunocompromised patients carries a bad prognosis which if left untreated rapidly progresses to cirrhosis (10% in 2 years) and end-stage liver disease (ESLD) [1, 13].

### 5.2. HEV Infection during Pregnancy

HEV infection during pregnancy is associated with increased risk of prematurity, abortion, low birth weight, perinatal mortality, fulminant hepatitis, and maternal mortality [6]. Maternal mortality rates are highest during the third trimester and have been reported to be as high as 25% [3]. Interestingly, in some countries such as Egypt, HEV infection during pregnancy is not associated with increased mortality. This phenomenon can likely be explained by the fact that the predominant genotype is less virulent [3].

## 6. Diagnosis

Clinical signs, symptoms, and laboratory findings often overlap with hepatitis from other etiologies and make confirmation of diagnosis difficult. Some experts suggest that testing for hepatitis E should be part of the diagnostic algorithm for all patients with acute or chronic hepatitis that cannot be explained by other causes [8].

Diagnostic methods are broadly classified into two types: direct and indirect. The direct methods detect the virus, viral proteins, or nucleic acids in blood and stool samples by immune-electron microscopy and RT-PCR. The indirect methods detect the anti-HEV IgM and IgG antibodies [1].

Detection of anti-HEV IgM is considered diagnostic for acute infection. The presence of IgG antibodies points out to previous exposure to HEV [4]. Anti-HEV IgM is detectable 4 days after the onset of jaundice and persists for up to 3–5 months [6]. Shortly after the appearance of IgM, IgG antibodies develop and peak at about 4 weeks after the onset of symptoms and persist for a variable period of 1 to 14 years after infection [6].

The detection of HEV RNA in biologic specimen (serum and/or stools) is the “gold standard” for the confirmation of acute HEV infection [9]. HEV RNA can be detected in stools 1 week before and up to 6 weeks after the onset of symptoms and in serum for 3-4 weeks from the onset of illness [4, 7].

The sensitivity of molecular tests for the detection of HEV RNA is dependent on how early the patient presents, timely collection of specimens along with its rapid transport, processing, and viral genotype inclusivity. Therefore, undetectable HEV RNA does not rule out recent infection [4].

PCR assays published so far have a high degree of performance variability. Therefore, World Health Organization (WHO) has recommended an international standard for HEV RNA detection and quantification that uses genotype 3a due to its worldwide distribution and its detection in chronic infections [3, 4, 6]. Another nucleic acid amplification technique, the loop-mediated isothermal amplification (LAMP) assay, has been developed for the detection of HEV RNA. The LAMP assay is quicker than real-time PCR and does not need special equipment, making it ideal for resource limited areas [3].

Insensitive and unspecific diagnostic tests for anti-HEV antibodies have made diagnosis challenging. In a study, in only 13.3% of the samples, anti-HEV IgM serology correlated to HEV polymerase chain reaction (PCR) positivity. This demonstrates an extremely low level of correlation with PCR-confirmed HEV infections [6]. Furthermore, false reactivity for anti-HEV IgM with Epstein-Barr virus (EBV) and cytomegalovirus (CMV), 33.3% and 24.2%, respectively, has been expressed in a study [6]. This is a clinically important consideration because these viruses form the differential diagnosis for acute non-A, non-B hepatitis. Nonetheless, recently developed “point-of-care” assays for anti-HIV IgM are simple, rapid, highly sensitive, and specific, ideal for resource-limited areas [3, 44]. Recently, novel efficient cell cultures have been generated for HEV3(4) and HEV4(3) that permitted the propagation of HEV in fecal and serum samples [3]. This discovery will be indispensable for extracting the infectivity titers of inocula in the future [3].

Anti-HEV-IgG and -IgM are fairly reliable methods of diagnosis in immune-competent hosts. However, they are frequently false-negative in immunocompromised host, which imposes a diagnostic challenge. RT-PCR is recommended to diagnose HEV infection in this subset of patients. In this setting, HEV RNA detection and quantification also has a role in monitoring response to antiviral therapy and determining the genotype of HEV involved [4, 5, 7, 9]. Nucleic acid amplification technology methods by RT-PCR have been demonstrated as having a higher sensitivity when compared to HEV IGM and HEV antigen. They are therefore better screening tests [45].

## 7. Treatment

Acute hepatitis E usually does not require treatment in immune-competent individuals. Data on treatment of HEV in immunocompromised, frequently chronic hepatitis is sparse, and, therefore, patient tailored therapy is the best option.

If HEV RNA persists for 3 months, then the patient is very unlikely to achieve spontaneous viral clearance without therapeutic intervention [3]. The most important step that should be considered is whether immunosuppression can be reduced. A study reports 25% HEV clearance rates by this strategy [46]. However, chances of transplant organ rejection greatly increase when immunosuppression is reduced. So, it can be rightly called a double-edged sword. Additionally, pegylated interferons have been used fairly successfully at times but are associated with significant side effects. They are better options in transplant recipients where reducing immunosuppression is not an option. Another somewhat promising option is ribavirin therapy. Ribavirin has been used to successfully treat severe acute hepatitis E patients with compromised immune systems [47]. Although there is no convincing data on it yet to make it a standardized option of HEV treatment, two French studies have shown virologic responses in 2 out of 2 and 4 out of 6 patients, respectively [48, 49]. However ribavirin is contraindicated in pregnancy. Liver transplant is the only option in patients who get fulminant hepatic failure.

## 8. Vaccination

Should one be infected by hepatitis E and recover, he or she will get protective immunity, with the courtesy of CD4 and CD8 T cells. Another way to induce immunity is via vaccination [50]. The HEV vaccine which is in the most advanced stages of development is HEV 239. It is a Chinese manufactured vaccine that has a 94–100% efficacy in a phase III trial conducted on more than 100,000 Chinese soldiers. Although it is based on the type 1 genotype, it works even against genotypes 1 and 4. Response to genotype 3 is not known. It is still in the development stage to be used worldwide but has been approved for use in China [51].

## 9. Conclusion

Keeping in view the history of identification of HEV, that is, a water-borne epidemic of acute hepatitis in a developing country, today, hardly few decades from its diagnosis, we can hardly limit it to “acute,” “hepatic,” “water-borne,” “epidemic,” or “developing” settings.

Recent identification of chronic HEV in immunosuppressed cases and its extrahepatic presentation manifests how limited our knowledge about HEV has been. One cannot stop wondering, “Do we know all about Hepatitis E?” We can no longer underestimate the importance of further research on the virus that had taken us off guard.

The fact that HEV is a common cause of acute hepatitis worldwide and recent appearance of many cases of acute hepatitis caused by HEV being misdiagnosed for causes such as drug-induced hepatitis, HEV testing should be considered among the first line when evaluating acute liver injury.

High mortality in pregnant females due to HEV and chronic HEV hepatitis in immunosuppressed cases and in those with underlying chronic liver disease expresses the urgent need of appropriate measures aimed at improving the current state of diagnosis and treatment of HEV.

Also, unexpectedly high seroprevalence in blood donors proclaims that emphasis be laid on consideration of screening of HEV in blood donors. And probably the first step would be to call attention to development of standardized diagnostic tests that have higher sensitivity and specificity and are cost-effective and commercially available.

## Figures and Tables

**Table 1:** Epidemiology and clinical features of hepatitis E virus [3, 6, 10, 20, 21].

Genotype	1	2	3	4
Host	Human; also isolated from pig	Human exclusively	Human, pigs, and other mammalian species	

Route of transmission	Fecal-oral; vertical transmission; zoonotic (genotype 1)		Zoonotic (usually swine, with humans being accidental hosts); environmental (shellfish, river); blood transfusion	

Geographical distribution	Mainly Asia and Latin America (Cuba, Venezuela, and Uruguay)	Mexico and West Africa	Worldwide	China, East Asia, Central Europe, and America

Epidemiological features	Causes epidemic outbreaks and sporadic cases in developing countries; occasionally in travelers returning from developing countries	Causes epidemic outbreaks and sporadic cases in developing countries	Causes sporadic autochthonous cases in developed countries	

Seasonality	Yes (outbreaks in flooding/monsoon)		No	

Clinical presentation	Mostly asymptomatic; acute self-limited hepatitis	Mostly asymptomatic; acute self-limited hepatitis	Varies from asymptomatic to acute self-limited hepatitis and may lead to chronicity in immunosuppressed cases (HEV3)	

Age	Adolescents and young (15–30 y)		Middle-aged and elderly (>50 y)	

Gender (M : F)	2 : 1		>3 : 1	

Prognosis	High mortality in pregnancy and in patients with underlying chronic liver disease	Fulminant hepatitis has not been noted in HEV2	Higher overall mortality rate relative to genotype 1; higher mortality among older adults	

Chronic infection	No		Yes	Yes

**Table 2:** Individuals at higher risk of acute liver failure from HEV infection [1, 3, 6, 7, 12, 22–24].

Pregnant women	
Individuals with preexisting liver disease	
Hepatitis B virus carriers	
Individuals with drug induced liver injuries	
Active alcohol abusers	
